# Gut microbiota derived bile acid metabolites maintain the homeostasis of gut and systemic immunity

**DOI:** 10.3389/fimmu.2023.1127743

**Published:** 2023-05-15

**Authors:** Xiaomin Su, Yunhuan Gao, Rongcun Yang

**Affiliations:** ^1^ Translational Medicine Institute, Affiliated Tianjin Union Medical Center of Nankai University, Nankai University, Tianjin, China; ^2^ State Key Laboratory of Medicinal Chemical Biology, Nankai University, Tianjin, China; ^3^ Department of Immunology, Nankai University School of Medicine, Nankai University, Tianjin, China

**Keywords:** gut microbiota, bile acids, deoxycholic acid, lithocholic acid, tolerogenic macrophages, regulatory B cells, regulatory T cells

## Abstract

Bile acids (BAs) as cholesterol-derived molecules play an essential role in some physiological processes such as nutrient absorption, glucose homeostasis and regulation of energy expenditure. They are synthesized in the liver as primary BAs such as cholic acid (CA), chenodeoxycholic acid (CDCA) and conjugated forms. A variety of secondary BAs such as deoxycholic acid (DCA) and lithocholic acid (LCA) and their derivatives is synthesized in the intestine through the involvement of various microorganisms. In addition to essential physiological functions, BAs and their metabolites are also involved in the differentiation and functions of innate and adaptive immune cells such as macrophages (Macs), dendritic cells (DCs), myeloid derived suppressive cells (MDSCs), regulatory T cells (Treg), Breg cells, T helper (Th)17 cells, CD4 Th1 and Th2 cells, CD8 cells, B cells and NKT cells. Dysregulation of the BAs and their metabolites also affects development of some diseases such as inflammatory bowel diseases. We here summarize recent advances in how BAs and their metabolites maintain gut and systemic homeostasis, including the metabolism of the BAs and their derivatives, the role of BAs and their metabolites in the differentiation and function of immune cells, and the effects of BAs and their metabolites on immune-associated disorders.

## 1 Introduction

Bile acid (BAs) are cholesterol-derived molecules involved in essential physiological processes including nutrient absorption, glucose homeostasis and regulation of energy expenditure (1). There are two main sites of BA biosynthesis, hepatocytes and gut microbiota. BAs are synthesized in the liver as primary BAs such as cholic acid (CA), chenodeoxycholic acid (CDCA) and their conjugated forms. A variety of secondary BAs such as deoxycholic acid (DCA) and lithocholic acid (LCA) and their derivatives, a large pool of bioactive molecules is synthesized in the intestine where they undergo bacteria-mediated transformation (2). BAs and their metabolites are abundant in the mammalian gut, and potentially distributed into other tissues and organs.

There exists a perfect immune system in different individuals, including innate immune cells such as macrophages (Macs), dendritic cells (DCs) and nature killer (NK) cells, and adaptive immune cells such as T cells and B cells. In addition to these cells, there also has a large amount of immune regulatory cells such as regulatory T cells (Treg cells), Breg cells, and innate immune lymphocytes (ILCs) to maintain local and systemic immune homeostasis. The differentiation and functions of these immune cells can be regulated by gut microbiota metabolites such as short-chain fatty acids (SCFAs) (3–5), tryptophan derived metabolites (6–8), and BA derivatives (9–12). BAs and their derivatives bind to multiple nuclear and cell surface receptors, which are expressed in the different immune cells such as Macs, DCs, MDSCs, Treg cells, Breg cells, ILCs, Th17 cells, CD4 Th1 cells, Th2 cells, CD8 cells, B cells and NKT cells. Each of BA and their derivatives has a different affinity for the receptor to which it can bind. While these receptors are bound and activated by different BA derivatives, they can affect the differentiation and function of different immune cells respectively. Understanding the effects of BAs and their metabolites on immune cells may elucidate a variety of disease states such as inflammatory bowel diseases, metabolic diseases, obesity, and other chronic inflammatory conditions. We here summarize recent advances in understanding the metabolism of BAs, the role of BAs and their derivatives in the differentiation and function of different immune cells, and the effects of BAs and their derivatives on immune-associated disorders.

## 2 BAs and their derivatives

### 2.1 BAs

BAs are the end-product of cholesterol metabolism (13, 14). The liver generates two primary BAs, i.e., CA and CDCA. The final products in the liver are mainly 3α-7α di-hydroxylated cholesterol derivatives, i.e., CDCA, and 3α-7α-12α-tri-hydroxylated derivatives, i.e., CA (14). These primary BAs in hepatocytes and/or in gut microbiota (15) are conjugated with glycine, taurine or other amino acids (15, 16). Then, the conjugated BAs are secreted into the intestine, becoming the substrate of an array of bacterial enzymes. This causes the generation of secondary BAs, i.e., LCA and DCA.

### 2.2 Secondary BAs and their derivatives

Secondary BAs DCA and LCA can be further modified into different derivatives by microbes (17). A range of oxo-, epi- and iso-derivatives of BAs is formed in the colon due to various dehydrogenation and epimerisation reactions in gut bacteria (18), such as 7-oxoCA, 7-oxoCDCA, 12-oxoCA and 12-oxoDCA (14). There also exist multiple forms of LCA derivatives such as allo-LCA, iso-LCA, isoalloLCA, 3-oxo-LCA, 3-oxoallo-LCA, and 3-ketoLCA (19, 20). In addition, the derivatives such as ursoDCA (UDCA) (21) and iso-DCA (9, 22) are also produced by 7α-hydroxysteroid dehydrogenase (7α-HSDH) and 7β-HSDH.

## 3 Effects of gut microbiota on BAs

**Figure 1:**
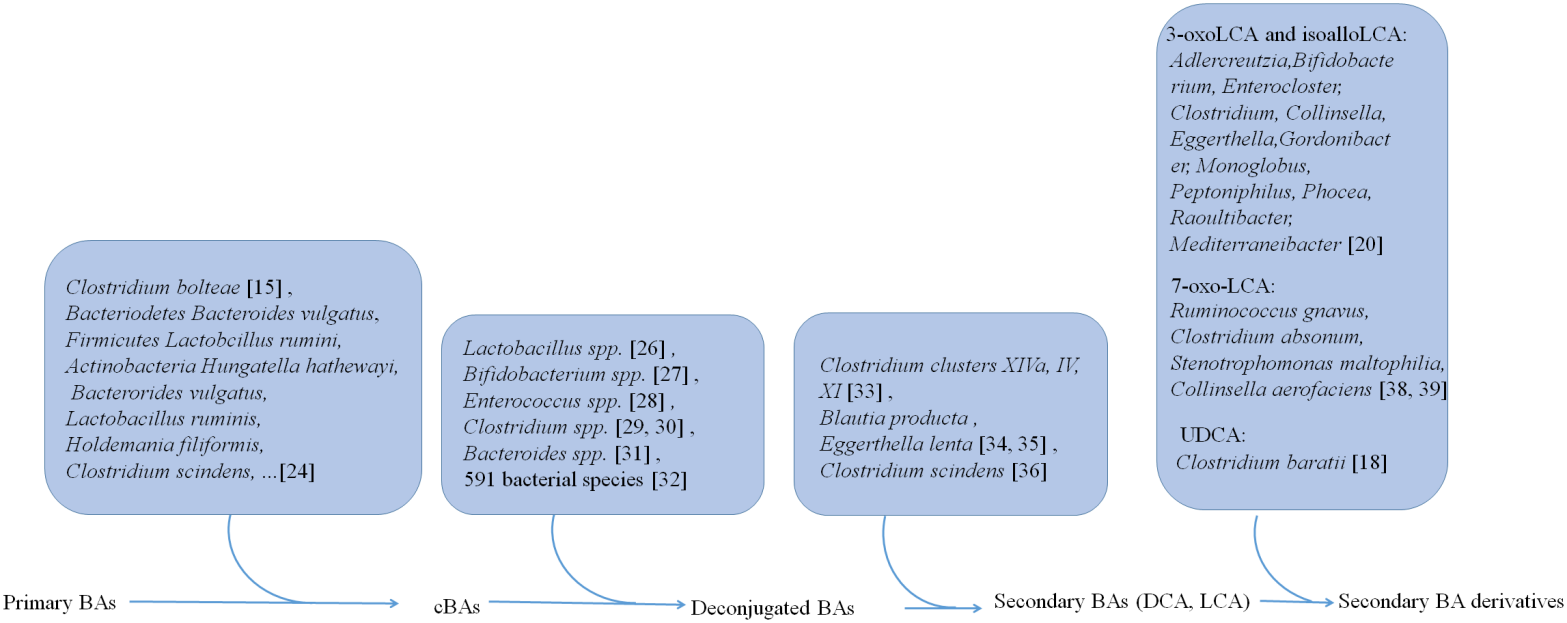
Gut microbiota is not only involved in the generation of conjugated BAs, but also plays a critical role in the transformation of BAs from conjugated BAs to deconjugated BAs and the generation of secondary BAs DCA and LCA and their derivatives.

Gut microbiota is not only involved in the generation of conjugated BAs but also plays a critical role in the transformation of BAs into other metabolites (**Figure 1**). In human, there have four distinct ways to transform BAs, including deconjugation, dehydroxylation, oxidation, and epimerization, which have been well reviewed (18).

### 3.1 Conjugation of BAs

Primary BAs can be conjugated with glycine and taurine in the liver. Then these conjugated BAs are released into the intestine *via* gallbladder (23). However, recent studies also show that gut microbiota such as *Clostridium bolteae* possesses an ability to conjugate BAs with phenylalanine, leucine, and tyrosine (15). 25 strains such as *Bacteriodetes Bacteroides vulgatus*, *Firmicutes Lactobcillus ruminis* and *Actinobacteria Hungatella hathewayi*, representing 24 species in the gut microbiota, can conjugate glycine to DCA, CDCA, or CA *in vitro* (24). 28 strains such as *Bacterorides vulgatus*, *Lactobacillus ruminis*, *Holdemania filiformis*, and *Clostridium scindens*, representing 27 species in the gut microbiota are capable of conjugating CDCA, DCA or CA to one or more other amino acids such as alanine, arginine and aspartate (24).

### 3.2 Deconjugated BAs

Liver derived conjugated BAs can be deconjugated in the small intestine by bile salt hydrolases (BSHs). These BSHs can be detected in gut microbiota (25) such as *Lactobacillus* spp (26)., *Bifidobacterium* spp (27)., *Enterococcus* spp (28)., *Clostridium* spp (29, 30)., and *Bacteroides* spp (31).. More recent studies show that BSHs can be found in 591 intestinal bacterial strains within 117 genera in human gut microbiota. Notably, 27.52% of these bacterial strains contains only BSH paralogs (32). These different phenotypes of BSHs exhibit different activity in the gut bacteria. BSH-T3, which is found in *Lactobaclillus*, shows the highest enzyme activity, whereas BSH-T5 and BSH-T6 mainly from *Bacteroides*, which have high percentage of paralogs, exhibit different enzyme and deconjugation activity (32).

### 3.3 Secondary BAs and their derivatives

After deconjugation, BAs can be converted into secondary BAs, i.e., DCA and LCA, and their derivatives by dehydroxylation, oxidation and epimerization.

Three distinct microbial 3α-, 7α-, and 12α- HSDHs, which result in oxidization and epimerization of specific hydroxyl groups on BAs can be found in gut microbes (33), such as *Clostridium clusters* XIVa, IV and XI. The bacteria such as *C. scindens*, *C. hylemonae* and *C. perfringens* are shown to produce enzymes capable of 3α-dehydrogenation. 3α-dehydrogenation also occurs in *Blautia producta* and *Eggerthella lenta* (34, 35). The BA transformations can also be carried out by 7-dehydroxylation in *Clostridium scindens in vitro* and *in vivo* (36). Recently, Funabashi et al. (37) showed that a set of six enzymes, which was necessary for conversion of CA to DCA, was engineered into a nonproducing bacteria, conferring production of DCA and LCA (37).

Paik et al. identified 12 human gut bacterial genera including *Adlercreutzia*, *Bifidobacterium*, *Enterocloster*, *Clostridium*, *Collinsella*, *Eggerthella*, *Gordonibacter*, *Monoglobus*, *Peptoniphilus*, *Phocea*, *Raoultibacter*, and *Mediterraneibacter*, which could convert LCA to 3-oxoLCA and isoLCA (20). In addition, both metabolites 3-oxoLCA and iso-alloLCA were absent in germ-free (GF) mouse models, also suggesting that these derivations were from microbiota (12). *Ruminococcus gnavus, Clostridium absonum, Stenotrophomonas maltophilia*, and *Collinsella aerofaciens* contribute to the ursoDCA pool *via* conversion of 7-oxo-LCA in an nicotinamide adenine dinucleotide (NADH) or nicotinamide-adenine dinucleotide phosphate (NADPH)-dependent fashion (38, 39). 7α-epimerization to UDCA also occurs in the gut bacterium members such as *Clostridium baratii* (18).

## 4 Regulation of BAs and their metabolites in the immune cells

**Figure 2:**
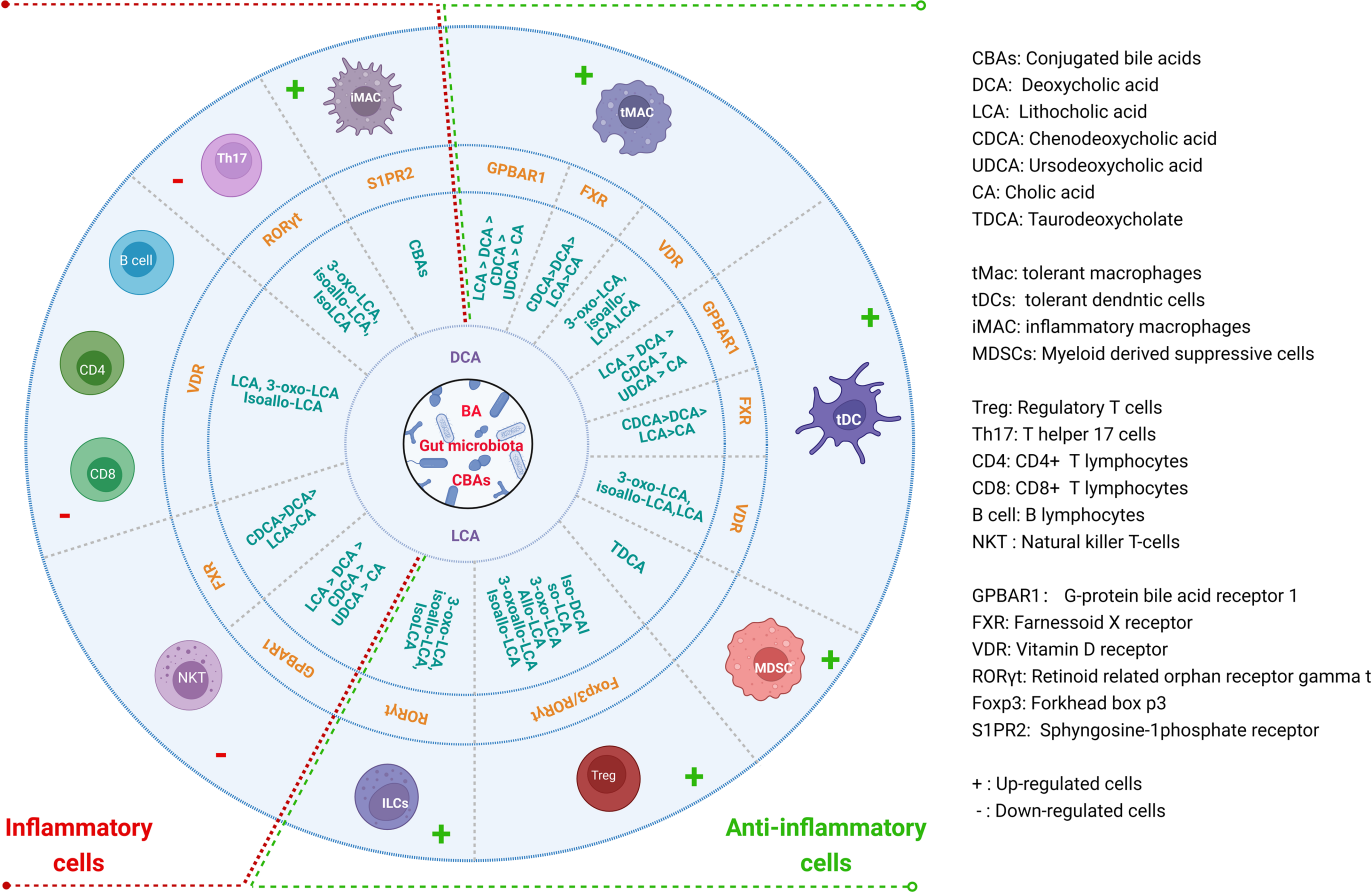
Regulations of BAs and their derivatives on the differentiation and function of immune cells for gut and systemic homeostasis. BAs and their derivatives not only promote the generation and function of anti-inflammatory cells, but also inhibit inflammatory cells through different receptors in the immune cells.

BAs and their metabolites can act on the receptors expressed in Macs, DCs, MDSCs, Tregs, Th17 cells, ILCs, CD4 cells, CD8 cells, B cells and NKT cells to modulate their differentiation and function for gut and systemic homeostasis (12, 14, 40–42) (**Figure 2**). These receptors include a range of nuclear receptors such as farnesoid X receptor (FXR), liver-X-receptor (LXR), pregnane X receptor (PXR), vitamin D receptor (VDR), retinoid related orphan receptor (RORγt), constitutive androstane receptor (CAR), and membrane receptors such as G-protein BA receptor 1 (GPBAR1) (Takeda G protein-coupled receptor 5 (TGR5)), sphingosine-1-phosphate receptor 2 (S1PR2), cholinergic receptor muscarinic 2 and 3 (CHRM2 and 3), and MAS related GPR (G-protein coupled receptor) family member X4 (MRGPRX4) (43), which have been reviewed by Biagioli et al. (44).

### 4.1 Myeloid derived cells

#### 4.1.1 Macrophages

Macrophages (Macs) can be mainly divided two subpopulations, inflammatory macrophages (iMacs) and immune tolerogenic macrophages (tMacs). IMacs (M1) are mainly involved in pro-inflammatory responses, whereas tMacs (M2) are mainly involved in immune suppressive responses. Intestinal Macs reside either within the lamina propria (LP) or the muscle layer. Muller et al. (45) have discussed recent advances in gut Macs. In the resting intestine, mature resident (immune tolerogenic) ly6c^low/-^CX3CR1^hi^MHC II^hi^ Macs from inflammatory Ly6c^high^ monocytes/Macs can express IL-10 and maintain intestinal homeostasis (46). Studies found that BAs and their metabolites can induce immune tolerogenic Macs. However, BAs, especially cBAs also cause inflammatory Macs. The contradicts in the effects of the BAs on the Macs are derived from different receptors expressed in the Macs. The majority of BAs-activated receptors such as FXR, TGR5, VDR, LXRs, PXR and S1PR2 have been detected in myeloid cells (10).

TGR5 is essential to maintain a tolerogenic phenotype of the Macs (10, 47, 48). Its activation can promote Mac polarization from the M1 (pro-inflammatory phenotype) to the M2 (immune tolerogenic phenotype) Macs, and reduce pro-inflammatory cytokines (49). TGR5 activation also blocks NLRP3-dependent inflammation such as lipopolysaccharide-induced systemic inflammation, type-2 diabetes-related inflammation and alum-induced peritoneal inflammation (50, 51). Secondary BAs DCA or LCA can function as endogenous inhibitors of NLRP3 inflammasome activation by activating TGR5 (52), which can cause a TGR5-cAMP-PKA-dependent ubiquitination of NLRP3 to inhibit its activation (53). The knockout of TGR5 in mice can accelerate LPS-induced inflammation in the liver and abolish the suppressive effects of TGR5 agonist on inflammatory cytokines (54). TGR5 natural ligands are LCA > DCA > CDCA > UDCA > CA (55). FXR is also essential to maintain a tolerogenic phenotype of the Macs as demonstrated in *FXR* KO mice (10). FXR can activate SOCS3, CYP450 and fibroblasts growth factor 19 (FGF19) to inhibit inflammation. In addition, FXR also activates SHP to inhibit NF-κB, AP-1 and NLRP3 (56–59), and is recruited to the iNOS and IL-1β promoters to stabilize the NCoR1 complexes, which can make these genes in the basal state (60). The assembly of NLRP3 inflammasomes is also suppressed by FXR, which physically interacts with NLRP3 and caspase-1 (52, 61, 62). In addition, PXR as a nuclear receptor also binds to LCA (55). PXR activation decreases the expression of IL6, TNFα, and IL8 mRNAs (42).

Notably, high cellular concentrations (≈100–500 μM) of BAs, particularly the hydrophilic secondary BAs, might function as danger-associated molecular pattern molecules (DAMPs) to cause a calcium-dependent activation of NLRP3 inflammasome (52, 62). However, this happens only while Macs are preactivated after exposure to endotoxin (52, 62). The hydrophobic primary BA such as ChenoDCA (CDCA) can induce NLRP3 activation and secretion of IL-1β by promoting ROS production and K^+^ efflux in Macs (63). Hao H et al. also found that BAs synergistically with ATP induced a prolonged calcium influx and activated NLRP3 (53). In addition, the conjugated BAs such as tauroCA (TCA) can activate S1PR2, which is shown to promote immune cell infiltration and inflammation in mouse models (64). Activating S1PR2 promotes caspase-11-dependent Mac pyroptosis and worsens *E. coli* sepsis (65). S1PR2 is also activated by the conjugated BAs to result in proinflammatory effects that can increase liver damage (66). S1PR2 deficiency significantly reduces cholangiocyte proliferation and cholestatic injury (64). Blockade of S1PR2 inhibits S1P-induced NLRP3 priming and inflammatory cytokine secretion (67).

#### 4.1.2 Dendritic cells

Dendritic cells (DCs) play a critical role in inducing protective adaptive immunity. However, DCs are also emerging as critical regulators of the immune responses (68). Secondary BA DCA suppresses LPS-induced expression of pro-inflammatory IL-1, IL-6, and TNFα in DCs (69), which can be rescued through DCA receptor TGR5 deficiency. The inhibitory effects of TGR5 are mediated through suppressing NF-κB by TGR5–cAMP–PKA signaling (69). BA-dependent TGR5 activation also induces the differentiation of human monocytes into IL-12 and TNF-α hypo-producing DCs *via* the TGR5-cAMP pathway (70). In addition, isoDCA can limit FXR activity in DCs and confer upon them an anti-inflammatory phenotype (9). The exposure of INT-747/obetiCA, which can activate FXR (10), greatly attenuates the differentiation CD14^+^ monocytes into mature DCs (71). A reduced number of activated DCs in the colon of mice administered with INT-747/obetiCA was also observed. In addition, VDR activation also inhibits the production of inflammatory cytokines, and the differentiation and maturation of DCs (72).

#### 4.1.3 Myeloid derived suppressor cells

Myeloid derived suppressor cells (MDSCs) play a key role in the immune suppression in some diseases, especially in cancer, and also have prominent role in tumor angiogenesis, drug resistance, and promotion of tumor metastases (73). Many pathogens, ranging from viruses to multicellular parasites, can promote the expansion of MDSCs (74). These MDSCs can be divided into monocytic and granulocytic MDSCs. The BA derivative TDCA can increase the number of granulocytic MDSCs in the spleen of septic mice (75).

### 4.2 Lymphoid derived cells

#### 4.2.1 CD4 T helper cells

There have multiple CD4 T helper (Th) cell subsets such as FoxP3^+^ regulatory T cells, RORγt^+^IL17^+^ Th17 cells, T-bet^+^IFNγ^+^ Th1 cells and Gata3^+^IL4^+^IL13^+^ Th2 cells. These CD4 Th cells play a critical role in maintaining the immune homeostasis of individuals. Studies have found that the differentiation and function of these cells can be regulated by BAs and their derivatives.

**Figure 3:**
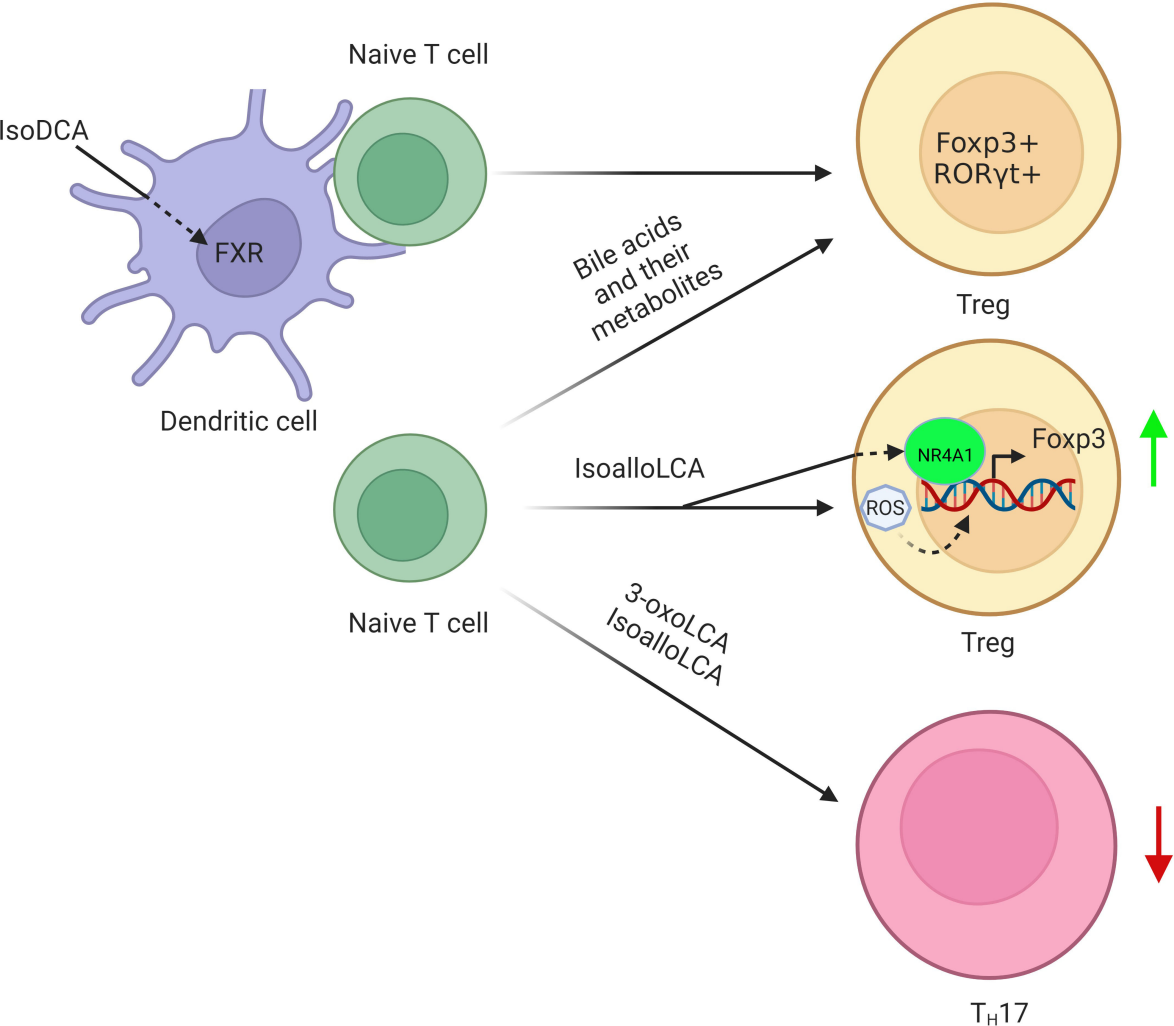
Regulation of BAs and their derivatives on the differentiation of Tregs. BA derivative isoDCA increases Foxp3 induction by diminishing DC immunostimulatory properties. IsoalloLCA promotes the differentiation of Treg cells through the production of mitochondrial reactive oxygen species, which lead to increased expression of Foxp3. Nuclear hormone receptor NR4A1 is also required for the effect of isoalloLCA on Treg cells. In addition, a distinct Treg population expressing the transcriptional factor RORγ is also induced in the colonic lamina propria by BAs and their metabolites.

1) FoxP3^+^T regulatory cells. FoxP3^+^T regulatory (Treg) cells express transcription factor Foxp3 (76, 77), and differentiate in the thymus or the periphery (78). BAs and their metabolites can affect the differentiation and function of Treg cells, which help protect against extracellular pathogens and maintain host immune tolerance, respectively (79). Indeed, secondary BAs such as isoalloLCA and isoDCA can promote the differentiation of Treg cells (9, 11, 12, 80, 81) (**Figure 3**). The isoalloLCA may be through the production of mitochondrial reactive oxygen species (mitoROS) to promote the expression of Foxp3 (11). Nuclear hormone receptor NR4A1 is also required for the regulation of isoalloLCA in Treg cells (80). Whereas the secondary BA derivatives isoDCA mediated Treg cells is through diminishing DC immunostimulatory properties (9). A distinct Treg population expressing the transcriptional factor RORγ can also be induced in the colonic LP by colonization with gut symbionts (81, 82). These RORγ^+^ Treg cells have a distinct phenotype (Helios^–^ and Nrp1^–^). Their accumulation is influenced by enteric factors derived from diet or commensal colonization (12, 81). In addition, VDR also drives T cell maturation facilitating the induction of T regulatory cells (83) and reduces Th17 cell formation (84).

2) RORγt^+^IL17^+^ Th17 cells. RORγt^+^IL17^+^ Th17 (Th17) cells cause autoimmunity and inflammation (85). The nuclear hormone retinoid-related orphan receptor γ (RORγ) is selectively expressed by Th17 cells, acting as a critical transcription factor for Th17 cell differentiation in chronic inflammation and autoimmune diseases (86). The BA metabolite 3-oxoLCA, which can directly bind to RORγ (11), inhibits Th17 cell differentiation by blocking the function of RORγ (11, 87). Similar to 3-oxoLCA, isoLCA also suppresses Th17 cell differentiation by inhibiting RORγ (20). A sulfated product of LCA, lithocholic acid 3-sulfate (CA-3-S) can also selectively inhibit Th17 cell differentiation by targeting RORγ (88). Thus, the inhibition of RORγ provides therapeutic benefits in the intestinal inflammation and reduces the frequencies of Th17 cells (89).

3) T-bet^+^IFNγ^+^ Th1 and Gata3^+^IL4^+^IL13^+^ Th2 cells. T-bet^+^IFNγ^+^ Th1 (Th1) and Gata3^+^IL4^+^IL13^+^ Th2 (Th2) cells can regulate appropriate cellular and humoral immune responses to pathogens and be involved in the progress of many diseases. Both IL-12 and IFN-γ make naive CD4^+^ T cells highly express T-bet and signal transducer and activator of transcription (STAT) 4 to differentiate to Th1 cells, while IL-4 makes naive CD4^+^ T cells highly express STAT6 and Gata3 to differentiate to Th2 cells. Through a VDR-dependent mechanism, the unconjugated LCA in physiological concentrations can inhibit the activation of human and mouse CD4^+^ Th1 cells, resulting in decreased TNFα and INF-γ production (90). VDR activation also promotes a shift from the Th1 to the Th2 phenotype through increased production of the transcription factors c-maf and Gata-3 (91). VDR can be activated by LCA and its metabolites such as 3-oxoLCA, 3-ketoLCA, LCA acetate, LCA propionate and iso-alloLCA. In addition, PXR activation also inhibit CD4 T cell proliferation *in vitro*. Liver puncture biopsy specimens from 34 matched patients before and after UDCA treatment showed the relationship between the infiltration of CD4 T cells and UDCA (92).

4) Innate lymphoid cells. Innate lymphoid cells (ILCs) are the importance in tissue homeostasis, morphogenesis, metabolism, repair, and regeneration. These cells can be divided into 3 groups, ILC1, ILC2 and ILC3 (93). In terms of function, ILC1s, ILC2s, and ILC3s mirror CD4^+^ Th1, Th2, and Th17 cells respectively (94). ILC3s can highly express BA receptors such as TGR5, FXR, and RORγt. The receptor RORγt is required for the generation of ILC3 (95).

#### 4.2.2 CD8^+^ cells

BA derivatives 24-NorursoDCA (NorUDCA) can reshape immunometabolism in CD8^+^ T cells and alleviate hepatic inflammation (96). TCA inhibits the response to IFNα therapy in the patients with chronic hepatitis B through suppressing CD8^+^ T and NK cell function (97). Cholestatic mice are featured with dysfunctional T cells response, as indicated by decreased sub-population of CD4^+^ and CD8^+^ cells and increased CTLA-4^+^CD4^+^ and CD8^+^ subsets (98). Transcription factor VDR activation also reduces the ongoing proliferation of CD8^+^ cells (99). PXR is expressed in human CD8^+^ T lymphocytes. PXR activation also inhibits CD8^+^ cell proliferation *in vitro.*

#### 4.2.3 B cells

B cells play an important role for immune response not only in antibody production but also in antigen presentation and cytokine production. BA receptor VDR activation reduces the proliferation of B lymphocytes (99), induces apoptosis of activated B cell apoptosis (100) and inhibits Ig production by B cells (101). Indeed, recent studies show that BAs can impair vaccine response, possibly *via* inhibiting post-class-switched memory B cell responses (102).

#### 4.2.4 NKT cells

NKT cells are an unusual population of T cells, which can recognize lipids presented by CD1d, a non-classical class I like molecule. These cells include two subtype, type I and II NKT cells, which pay a critical role in tumor immunity. Type I NKT cells generally promote tumor immunity; whereas type II NKT cells suppress it. But, type I NKT cells can also induce immunosuppressive cells such as Treg. BA receptor FXR activation in NKT cells results in a profound inhibition to produce osteopontin, a potent pro-inflammatory mediator along with IL-1β and IFN-γ (60). TGR5 agonists induce NKT cells polarization toward IL-10 secreting type I NKT cells and significantly expand the subset of IL-10 secreting type II NKT cells (103). Recent studies show that gut microbiota-mediated BA metabolism can regulate liver antitumor immunity *via* controlling an accumulation of NKT cells (104).

## 5 BA metabolites and immune-associated disorders

**Figure 4:**
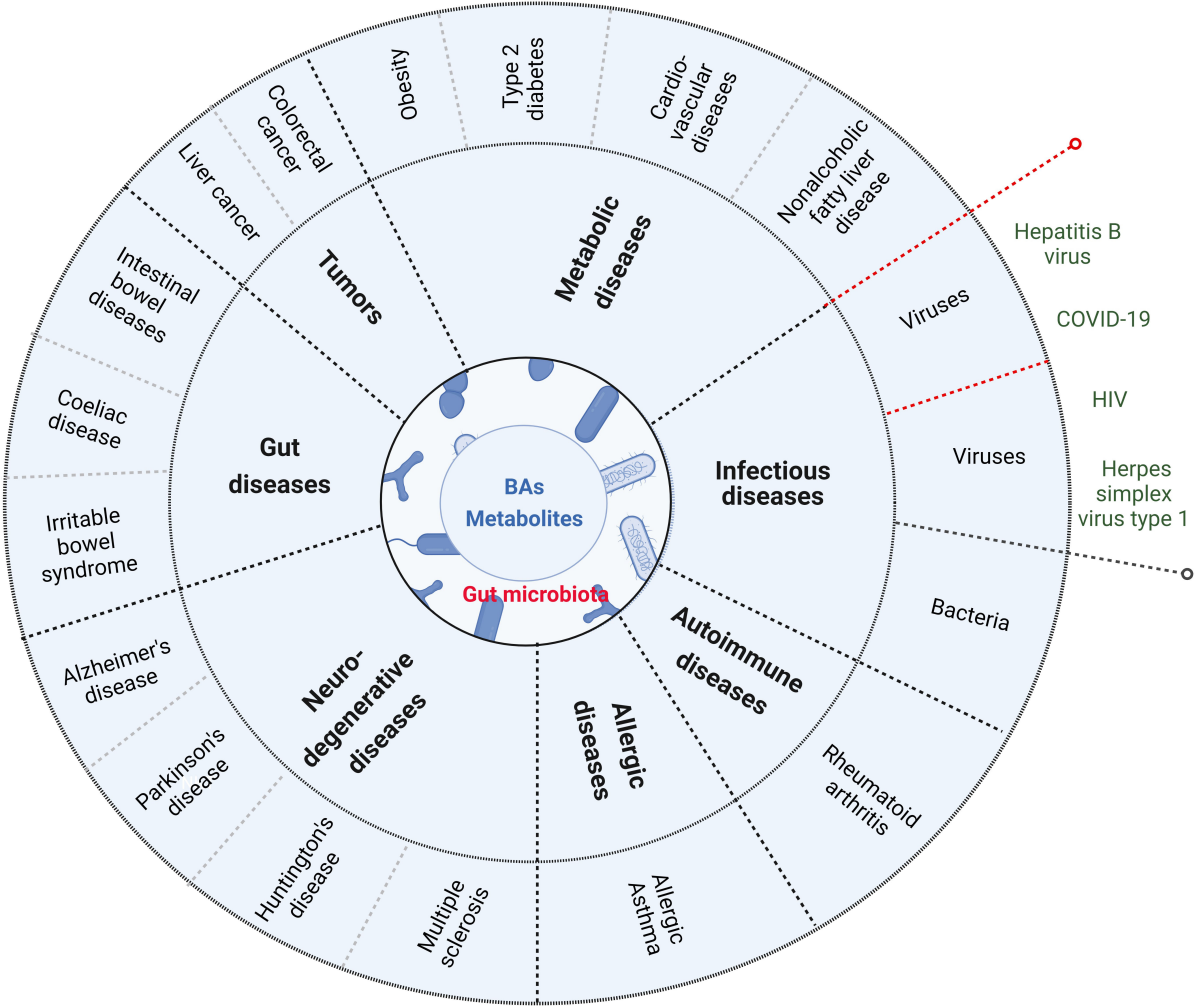
BAs and their metabolites are related to the occurrence and development of immune-associated disorders such as gut diseases (inflammatory bowel disease, coeliac disease and irritable bowel syndrome), metabolic diseases (obesity, type 2 disbetes, cardiovascular disease and nonalcoholic fatty liver disease), tumor (such as colorectal cancer and liver cancer), neurodegenerative disease (Alzheimer’s disease, Parkinson’s disease, Huntington’s disease and Multiple sclerosis), allergic diseases, autoimmune diseases and infections (such as bacterium infection). Red dotted lines indicate that BAs and their metabolites in virus infection can also promote innate immunes.

BAs and their metabolites play an important role in maintaining the homeostasis of local and system immunes. Damages of the homeostasis are related to the occurrence and development of immune-associated disorders such as gut diseases, metabolic diseases, tumors, neurodegenerative diseases, allergic diseases, autoimmune diseases and infectious diseases (**Figure 4**) (105).

### 5.1 Gut inflammation associated diseases

Gut diseases such as inflammatory bowel diseases (IBDs), coeliac disease and irritable bowel syndrome are gut inflammation associated diseases. Intestinal bowel diseases (IBD), including Crohn’s disease (CD) and ulcerative colitis (UC), are chronic relapsing disorders (14). The effects of BAs on IBD have been reviewed (10, 44, 106). Accumulating evidences have shown that the gut microbiota plays a pivotal role in maintaining intestinal homeostasis. There exist decreased microbial diversity and abnormal microbial composition in the patients with IBD (99), which are characterized by increased phyla *Proteobacteria* and *Fusobacteria* (mainly *Fusobacteria varium* in UC and *Fusobacteriaceae* in CD patients), and reduced phyla *Firmicutes* (107–111). Since the majority of BSH expressing bacteria is members of *Firmicutes* phylum (112), these changes might impact on BA metabolism, which is related to the occurrence and development of IBD (113).

Recent metabolomics has revealed a consistent defect in the BA metabolism, which is companied with an increase in primary BAs and a reduction in secondary BAs in the patients with IBD (114, 115). Vantrappen et al. first demonstrated that the decrease in the BA pool size is inversely correlated with the Crohn’s disease activity index (116). There also exhibit a severe reduction in fecal secondary BAs such as DCA and LCA, and an increased abundance of primary bile acids such as CA and CDCA in active patients with IBD (115). The levels of 3-oxoLCA and isoLCA are also significantly reduced in the patients with IBD (20). Similar findings with increased primary BAs and their conjugated forms, and reduced secondary and unconjugated BAs (117) are also observed in paediatric patients with IBD (118). In addition, a reduction of LCA also impacts on activation of VDR, that is an anti-inflammatory receptor in macrophages. The patients with IBD are also characterized by an increased 3-sulfated DCA and LCA in the feces (119), suggesting that in addition to BSH-dependent deamidation, other biotransformations such as sulfatation might also be impaired in patients with IBD (119). Thus, the supplementation of secondary BAs may be a potential strategy for the therapy of the patients with IBD.

Primary and secondary BAs are identified as signaling molecules acting on a family of cell membrane and nuclear receptors such as TGR5, FXR, PXR and VDR, which are highly expressed in the gastrointestinal tract. Studies have demonstrated that both BA receptors FXR and TGR5 are essential to maintain a tolerogenic phenotype of intestine immune. Ablating these receptors can promote the polarization of intestinal T cells toward a pro-inflammatory phenotype (14). *FXR* or *TGR5* KO mice are prone to develop an exaggerated inflammatory response upon exposure to dextran sodium sulfate (DSS) or trinitrobenzene sulfonate (TNBS) (120). Upon BA activation, FXR controls expression of genes, which can limit the inflammatory responses. *FXR* KO naïve mice are characterized by intestinal inflammation with increased expression of pro-inflammatory cytokines as compared to wild type (WT) mice (120). In addition to FXR, PXR is also involved in IBD. Human PXR activation represses intestinal immune response in a NF-κB dependent manner (121). Compared with WT mice, DSS induced colitis was more severe in *PXR* KO mice (122), which could be protected by pregnenolone 16α-carbonitrile (PCN, a human PXR agonist) (122). Notably, several studies showed that PXR polymorphisms had no markedly effects on the risk of IBD (123). Another nuclear receptor VDR can also be activated by the secondary BA LCA and/or its metabolites 3-oxoLCA and iso-alloLCA (12). Studies found that VDR plays a beneficial role in patients with IBD (124). Its polymorphisms are related with susceptibility to IBD (125). In mouse model of colitis, *VDR* KO can exacerbate the symptoms in *IL-10* KO mice, whereas vitamin D supplementation improves the symptoms. Intestinal epithelial cells-specific *VDR* KO mice showed a more severe colitis than WT mice (126). Taken together, these receptors may provide new perspectives on the treatment of intestine diseases such as IBD.

### 5.2 Metabolic diseases

Metabolic diseases such as obesity, type 2 diabetes (T2D), cardiovascular disease and non-alcoholic fatty liver disease (NAFLD), are generally considered a chronic inflammatory disease (127, 128). Altered bile acid metabolism can contribute to these chronic inflammatory diseases (129). BAs and their derivatives are valuable therapeutic agents for treating these inflammatory metabolic diseases (129).

Obesity is mainly induced by the disequilibrium of energy intake and energy expenditure, which results in metabolic disorders and chronic low-grade inflammation. UDCA supplementation can control diet-induced obesity in prenatally malnourished mice (130). Dietary acetic acid suppress high-fat diet-induced obesity in mice by altering taurine conjugated bile acids metabolism (131). Watanabe et al. reported that the administration of BAs to mice increased energy expenditure in brown adipose tissue (BAT), preventing obesity and resistance to insulin (132). The proportion of non-12-OH bile acids, including HCA, HDCA, glycohyodeoxycholic acid (GHDCA), UDCA, GUDCA, and CDCA in total bile acid is significantly lower in people with high body mass index (BMI), indicating that non-12-OH bile acids may contribute to the process of obesity (133). In the individuals with obesity, T2D and NAFLD, which are characterized by recruitment of immune cells, abnormal production of cellular inflammatory cytokines and acute phase reactants, and activation of inflammasomes, are associated with dysregulation of BA homeostasis (127, 134). Recent studies suggested that size and/or composition of BA pool changed in patients with T2D, and found that BAs and their derivatives improved T2D by reducing the levels of inflammatory cytokines (135). BA metabolism is also altered in patients with hepatic steatosis and glucose and lipid dysmetabolism (136). Dysregulation of BA metabolism was linked to steatosis, inflammation, and fibrosis in patients with NAFLD (137). Intervention of BAs could effectively control and prevent obesity and NAFLD (132, 138). Studies from animal models and human patients have found that NAFLD disease progression is closely associated with BA dysregulation (139–143).

Inflammation plays an important role in the development and progression of cardiovascular diseases (CVDs). Hypertension and hyperlipidemia, the key risk factors of CVDs, are related to inflammation in the heart and vessels (144). The signaling pathways mediated by immune and inflammatory mediators have been implicated within the atherosclerotic lesion (145). A growing number of studies have shown a strong relationship between gut microbiota and CVDs such as coronary atherosclerosis, hypertension and heart failure (146). High fiber diet significantly improved cardiac function through modulating the composition of intestinal flora and the production of metabolites production, including the biosynthesis of bile acids and linoleic acid metabolism (147). Dietary mannan oligosaccharides can increase fecal BA excretion and decrease atherosclerosis development (148).

### 5.3 Tumors

BAs have been considered as pro-carcinogenic molecules (149, 150). Studies have also implied the involvement of BAs in colorectal, gastric, hepatocellular, pancreatic, breast, prostate and ovarian cancer (149). However, inflammation play a decisive role in inducing tumorigenesis, promoting tumor development, tumor invasion and migration (151). Human epidemiological evidence has confirmed the close relationship between chronic inflammation and tumorigenesis (152) such as that inflammation is a common medical complication in colorectal cancer (CRC) patients, which plays significant roles in tumor progression and immunosuppression (153). Some epidemiological studies have shown an association between fecal and serum BAs and CRC (154). TGR5 activation by UDCA and LCA can exert anti-inflammatory responses through TLR4 activation or by reducing pro-inflammatory cytokine production in the colon that can decrease the frequency of developing CRC (155). Altered BA metabolism also promoted helicobacter pylori-induced inflammation-driven gastric carcinogenesis (156). Remarkably decreasing percentages of serum conjugated DCA were closely associated with hepatocellular carcinoma (HCC) (157).

### 5.4 Neurodegenerative diseases

Various studies have shown the role of neuro-inflammation in the occurrence, diagnosis, and treatment of neurodegenerative diseases. Neuro-inflammation can trigger the formation of other factors responsible for causing several neuronal diseases including Alzheimer’s disease (AD), Parkinson’s disease (PD), Huntington’s disease (HD), multiple sclerosis (MS), ischemia, and several others (158, 159). Parkinson disease (PD) is a progressive neurodegenerative disease that affects peripheral organs as well as the central nervous system and involves a fundamental role of neuro-inflammation in its pathophysiology. There is increasing evidence for inflammation as a determinant in the pathogenesis of Parkinson’s disease (160). UDCA and TUDCA have shown neuroprotective properties in these neurodegenerative diseases (161). TDCA is also as a potential therapeutic tool in neurodegenerative diseases (162).

### 5.5 Rheumatoid arthritis

Rheumatoid arthritis (RA) is an autoimmune disease characterized by joint destruction, synovitis, and pannus formation. Additional proinflammatory cytokines, such as IL-7, IL-17, IL-21, IL -23, GM-CSF, IL-1β, IL-18, IL-33 and IL-2 are involved in the pathogenesis of RA (163). Elevated levels of primary BAs have been found in the feces of some RA patients, which can be used to predict RA arthritis severity (164). Secondary BAs such as DCA and LCA can suppress macrophage cytokine production *via* FXR (165).

### 5.6 Allergic asthma

Obesity is a risk factor for the development of asthma and is associated with worsening symptoms and poor asthma control (166). Altered bile acid profiles have been reported in asthmatic patients. GCA, GDC, TCDC and taurocholate increased with asthma, compared to healthy individuals (167).

### 5.7 Infectious diseases

Bacterium and virus infection can cause inflammation. BAs are associated with infectious diseases such as *Clostridioides difficile* or *Salmonella* Typhimurium infection (105). BAs regulate immune responses upon ligation of these two receptors FXR and TGR5, which are located at the interface of the host immune system with the intestinal microbiota (10). However, studies have also reported that BAs activated several key innate signaling pathways to potentiate antiviral immunity (10). The intestinal regionalization of acute norovirus infection is regulated by the microbiota *via* bile acid-mediated priming of type III interferon (168, 169).

## 6 Conclusion

There are multiple forms of BAs such as conjugated and deconjugated primary BAs, secondary BAs DCA and LCA, and their derivatives. While primary BAs are generated in the liver, four distinct ways, including deconjugation, dehydroxylation, dehydrogenation and epimerization are used to transform primary BAs into secondary BAs and their derivatives by gut microbiota. These primary and secondary BAs and their derivatives can act on the receptors expressed in Macs, MDSCs, DCs, Tregs, Th17 cells, ILCs, CD4 cells, CD8 cells, B cells and NKT cells to modulate their differentiation and function, which can affect both the innate and adaptive immune responses for homeostasis. In addition, dysregulation of BA homeostasis is also found in the inflammation associated disorders such as IBD.

BAs have been used therapeutically in China for over 2500 years (170). Currently, the Food and Drug Administration (FDA) has approved a formulation of UDCA, Ursodiol, which has vast beneficial effects such as anti-inflammatory (171). It has been used to treat a variety of diseases such as cholesterol gallstones, primary biliary cirrhosis, primary sclerotic cholangitis, nonalcoholic fatty liver disease, chronic viral hepatitis C, recurrent colonic adenomas, cholestasis of pregnancy, and recurrent pancreatitis (171). With understanding of BAs and their metabolites on the local and systemic immunes, more precise therapy based on BA metabolites will be used in inflammation-associated diseases.

## Author contributions

XS, YG made the figures and wrote the original manuscript. RY improved and wrote the final manuscript. All authors contributed to the article and approved the submitted version.
